# Image Analysis: A Versatile Tool in the Manufacturing and Quality Control of Pharmaceutical Dosage Forms

**DOI:** 10.3390/pharmaceutics13050685

**Published:** 2021-05-10

**Authors:** Dóra Farkas, Lajos Madarász, Zsombor K. Nagy, István Antal, Nikolett Kállai-Szabó

**Affiliations:** 1 Department of Pharmaceutics, Semmelweis University, Hőgyes Str. 7, H-1092 Budapest, Hungary; farkas.dora1@pharma.semmelweis-univ.hu (D.F.); antal.istvan@pharma.semmelweis-univ.hu (I.A.); 2 Department of Organic Chemistry and Technology, Budapest University of Technology and Economics, Műegyetem rakpart 3, H-1111 Budapest, Hungary; madlajos96@gmail.com (L.M.); zsknagy@oct.bme.hu (Z.K.N.)

**Keywords:** image analysis, pharmaceutical dosage form, foam, particle size, shape parameters

## Abstract

In pharmaceutical sciences, visual inspection is one of the oldest methods used for description in pharmacopeias and is still an important part of the characterization and qualification of active ingredients, excipients, and dosage forms. With the development of technology, it is now also possible to take images of various pharmaceutical dosage forms with different imaging methods in a size range that is hardly visible or completely invisible to the human eye. By analyzing high-quality designs, physicochemical processes can be understood, and the results can be used even in the optimization of the composition of the dosage form and in the development of its production. The present study aims to show some of the countless ways image analysis can be used in the manufacturing and quality assessment of different dosage forms. This summary also includes measurements and an evaluation of, amongst others, a less studied dosage form, medicated foams.

## 1. Introduction—Image Analysis (IA) for Pharmaceutical Purposes

Pharmacopoeias describe visual methods for several purposes, such as to determine the absence or presence of visible particles, for the evaluation of chromatograms (identification of the principal and secondary spots), comparison of the relative retentions, melting point determination, or even for microbiological control purposes. Visual inspection (VI) has an essential role in the manufacturing of injectable medicinal products, as they cannot contain any sub-visible particles—all containers have to be visually inspected either manually or with an automated system. Contrary to the US Pharmacopeia (USP), both the European and the Japanese pharmacopoeias specify the circumstances for the tests of visible particulate contamination—the type and intensity of illumination, the backgrounds, and also the time of inspection [1,2,3]. Acknowledging the importance of VI, Madsen et al. published a proposal to revise the general chapter of injections in the USP to decrease the risk of not noticing unintended particulate matter due to the human visual performance [4]. In 2013, the ECA Visual Inspection Group was formed to assemble knowledge and practical solutions in a guidance paper on the VI of parenteral preparations. Therein, the best practice for carrying out visual inspection of parenteralia is highlighted. The paper gives instructions for both manual and automated inspection, specifying the criteria for the workplace, personnel, operation, and also validation. It also classifies defects and gives concrete advice on the evaluation and training of the inspection staff [5,6].

The origin of mathematical morphology dates back to 1964 when two engineers, Georges Matheron and Jean Serra set the basics for the analysis of binary images. They studied the geometry of porous media and considered the matrix and the pores as complements, and consequently used basic operations (unions, intersections, complementation) for the processing of binary image objects. Mathematical morphology, which is based on algebra and integral geometry, is an essential part of image processing [7,8].

**Figure 1:**
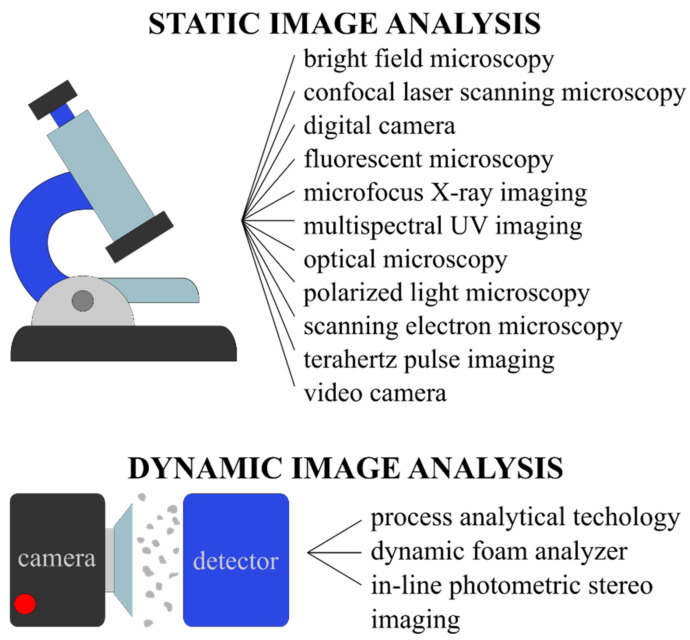
Comparison of image acquisition methods for static and dynamic image analyses.

Image analysis can be categorized as static or dynamic based on the image acquisition method used (Figure 1). Static image analysis is performed when the particles are still and motionless, such as in optical or scanning electron microscope images. It has to be noted that for this type of analysis the limited count of measurements along with the sampling size or segregation can lead to result inaccuracies, and the results are also highly influenced by sample preparation. On the other hand, dynamic image analysis is carried out on samples where the particles are dispersed in a gas or liquid medium or on a conveyor, and a high-speed matrix or line camera is used for image capturing. Dynamic IA results are repeatable but in shorter times they stand out in robust statistics. The importance of performing particle size analysis using image analysis methods is also proven by the fact that ISO guidelines also cover the topics of both static and dynamic methods [9,10,11].

Image analysis is extensively used in the pharmaceutical sciences for a wide variety of purposes (see the graphical abstract). It is not only used for the classification of final products, but can be applied as an important part of suitable process analytical technology (PAT)-based methods for in-process control (IPC) purposes. PAT enables the in-line analysis of pharmaceutical manufacturing even in case of continuous manufacturing. Machine vision (MV) opens new windows of opportunity in this field as it enables more than the exhaustive analysis of the product. Processes like crystallization, milling, emulsification, fluidization, granulation, tableting, and coating can be followed and the accidental disturbances during these processes can be detected in real time [12]. Machine vision has been used for simple tasks because it can provide descriptive analysis along with reliable detection rates. Deep learning is a further improved part of MV that also gains information (learns) from the inputs, but with the creation of a neural network similar to that of a human brain, it is also capable of predictive analysis. This way it can not only identify but also classify the defects [13].

In this summary, studies are presented where the analysis of images taken with different imaging techniques was used for the characterization and examination of various pharmaceutical dosage forms. Additionally, the review has been supplemented with original measurements for a less-studied dosage form, medicated foams.

The different dosage forms tested were divided into larger groups according to their state of matter. In addition to the exact form, the tables (see later) also show the applied imaging procedure and the software used by the authors to conduct the studies, as the effect of the software and the shape factor algorithms used must be taken into account [14]. It is also important to note, that the different software may use diverse terminology for the same shape descriptor or another formula for its calculation [15]. All in all, the pharmaceutical dosage forms can be described with various descriptive parameters (e.g., shape, texture, size, frequency) that may demand a special or dedicated software suitable for the image analysis of the sample (like in case of medicated foams).

## 2. Image Analysis of Pharmaceutical Dosage Forms

For the study of pharmaceutical dosage forms, the pharmacopoeias specify tests with which they always have to comply. Further to these methods, there are non-pharmacopoeial ones as well that may supplement further information on the parameters studied. Image analysis provides a new, more significant input in the study of pharmaceutical dosage forms, notably due to the advantages it has over the conventional methods (as shown in Table 1) listed in the pharmacopeias.

The table does not show that many of the above-mentioned image analysis solutions can be implemented with commercially available patented equipment. Thus, e.g., PSD can be easily measured using in-line or on-line mode with equipment based on dynamic image analysis [22,23]. Today, it is also possible for automatic visual inspection and grading of tablets, capsules, and softgels [24]. Furthermore, patented machines can be found for foamability and foam stability testing too [25]. In the following, the use of IA for the quality control of pharmaceutical dosage forms will be discussed more thoroughly, based on the state of matter. Brief summaries on the examined parameters (aim of measurement), the image acquisition methods, along with the used software are shown for solid (Table 2), semisolid (Table 3) and liquid (Table 4) formulations are also presented.

### 2.1. Solid Dosage Forms

#### 2.1.1. Powders

Barely any manufacturing process of pharmaceutical dosage forms is feasible without the involvement of powders of any form. The powder mixture containing the active ingredient can be the end product, as a dosage form (e.g., powdered powder, a powder mixture in a sachet), or even an intermediate that can be used, for example, for granulation or direct compression.

Particle size and characteristics are of great importance as not only do they influence the processing conditions but also numerous crucial properties of the final preparation from dissolution to bioavailability. This is the reason for the increasing interest in the extensive understanding of the used materials and their impact upon the processes [26]. It is important to note that the microscopic and conventional digital images are just two-dimensional, this way only a projection of the particle can be investigated. As the particles are usually stably located on the largest part of the surface, the largest diameter is the most relevant parameter to determine [27]. Additionally, studies on the effect of the number of the examined particles have shown that with the increasing sample size the standard deviation decreases. This shows the pertinence of the sample size required to obtain results with certain accuracy [27,28,29]. Overlapping particles or agglomerates may cause deviance in the results, but with deep learning-based image segmentation this problem can be eliminated [30]. Image analysis is already of wide-ranging use in powder characterization. Optical microscopy allows for the examination of micronized bulk powders. When choosing the most suitable sample preparation method, it is important to take the width of the particle size distribution and the contribution of particles smaller than the resolution limit to the total particle volume into account. Zingerman et al. validated a computerized image analysis system for particle size analysis of micronized bulk powders by comparing the optical microscopic results to scanning electron microscopy (SEM)-based analysis [31]. Kelly et al. compared IA data with particle size data obtained using a more traditional, laser diffraction method. Their aim was to evaluate the results of the measurements on non-spherical particles with different morphological characteristics and found that the outcomes can be successfully correlated [32]. Boschetto et al. worked on a digital-image-processing-based method that characterizes the 2D morphology and morphometry of granular substances while giving a 3D shape description [33].

The mixing or blending of powders is a key operation in the pharmaceutical industry as the quality of mixing influences the content uniformity as well as the quality of the final product. The mixing time is affected by several parameters, including the particle size and the type of the mixer. To predict the required time for mixing pharmaceutical powders, Mahdi et al. used a new approach. They coupled the artificial neural networks with image analysis for the characterization of the homogeneity of powder mixtures [34]. Berthiaux et al. developed an IA-based method for the measurement of powder homogeneity of loose materials and proposed their real-time principal component analysis as an on-line methodology adaptable to other techniques [35]. The degree and rate of segregation in polydisperse systems during fluidization can also be monitored with the help of IA [36,37].

#### 2.1.2. Dry Powder Inhalers

Dry powder inhalers (DPI) are frequently used in the inhalation therapy of common chronic airway diseases like asthma or chronic obstructive pulmonary disease (COPD). Overcoming the disadvantages of pressurized metered-dose inhalers—difficulty of application, environmentally hazardous propellants, lack of indicator to inform about the remaining medicine—DPIs gained better patient acceptance than their predecessors [38].

Aerodynamic particle size distribution is a key property to estimate the orally inhaled drug content and consequently important in the development of inhalation devices for the specific drugs. Cascade impactors are official in the European Pharmacopoeia, but other than being expensive they are also highly time-consuming [39]. As an alternative, IA-based methods were developed [40,41]. Fishler et al. used IA for particle setting velocity estimations to obtain the required PSD data [41,42].

As the importance of DPIs rose, specific analytical methods were evolved. Malvern Panalytical Ltd. developed Morphologi, a fully automated static IA-based system that can be used for the assessment of particle characteristics in details. Due to continuous improvements, the company combined Morphologi with Raman spectroscopy in an integrated platform, thus component-specific morphological descriptions are now provided [43,44].

Dry powders can also be applied in the form of aerosols with suitable dispensers as these devices use mechanical force to break the agglomerates or aggregates [1].

#### 2.1.3. Granules

Granules are solid dry powder aggregates containing one or more active pharmaceutical ingredients (APIs) and intended for direct oral administration or further processing. As independent dosage forms, they can be of single-dose (in sachets) or multidose administration (suitable measuring device provided). The European Pharmacopoeia categorizes them into four groups: effervescent, coated, gastro-resistant, and modified-release granules [1].

The particle shape and size influence the flow behavior of granules, therefore these characteristics are of great importance when it comes to packing. Sandler and Wilson used the PSD data for the prediction of flowability and packing behavior of several different pharmaceutical particulate systems [45].

#### 2.1.4. Pellets

The concept of multiparticulate dosage forms was introduced in the 1950s. The importance of pellets is supported by the fact that the first approved modified-release formulation was a capsule containing pellets. The product made by Smith, Kline & French (now GlaxoSmithKline, Brentford, UK), Dexedrine^®^ Spansule^®^, was able to provide therapeutic levels for up to 12 h. The production and commercialization of the formulation containing dexedrine-containing layered sugar granules began in 1952 and it is still marketed in the United States of America after a minor color modification in 1996 [46,47,48].

Owing to the numerous advantages of multiunit dosage forms, the extensive research on pelletization optimization resulted in the development of investigation methods [49]. Image analysis is often used for pellet analysis as it is a simple and cost-effective method that enables the size and shape description in one step. Several different shape descriptors can be calculated for more accurate characterization [29,50]. IA is often implemented in-line for pellet manufacturing. Treffer et al. carried out hot-melt extrusion with hot-die face pelletizing and used an in-line photometric stereo imaging system for the monitoring of pellet shape and size distribution [51]. Almeida-Prieto et al. examined not only the particle images but also simulated images to evaluate two newly defined form factors: Vr, which characterizes the radius variability, thus indicating the shape, and Vp, which enumerates the acircularity of the perimeter, consequently providing information on surface roughness [52].

**Figure 2:**
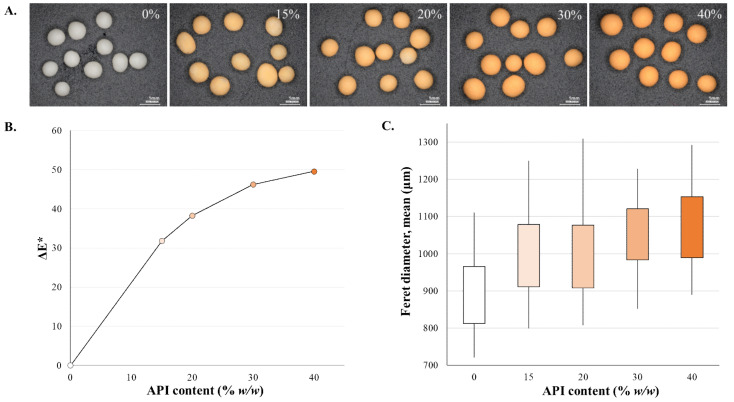
(**A**) Color change of pellets during coating progress, (**B**) the color analysis results, and (**C**) the size analysis results (*n* = 200) (evaluation of own experimental data).

Coating pellets can provide several advantages by altering the taste, the site of dissolution, and also the release kinetics. Due to the low surface area–volume ratio and the shape of the pellets, they are ideal for film coating [53]. The quality and the thickness of film coating are the main influencers of the drug release rate of sustained-release pellets [54]. For the at-line or in-line analysis of the coating process, image analysis is frequently used by itself or combined [55,56,57,58]. Upon incorporating a red water-soluble dye into the transparent coating, Kennedy and Niebergall split the digital color images into red, green, and blue (RGB) channels to enhance the images and enable the determination of optical density that correlates with the coating levels. This rapid and objective method is not only cost-effective but it is also exceptionally precise [21]. A similar approach was used for the evaluation of pellet coating thickness when inert pellet cores (microcrystalline cellulose, diameter: 710–1000 μm) were coated with an ibuprofen-containing dispersion (colored with sunset yellow dye). As the coating progressed (the percentage of the coating increased), the change in the color and size was noticeable and measurable (Figure 2A). The color change was analyzed from digital microscopic (Keyence VHX-970F; Keyence Corp., Osaka, Japan) pictures (2048 × 1536 pixels; 10 pellets/sample) and the L*a*b* (or CIELab) color space was used for the numeric description. As the orange color intensifies, both the a* (positive values toward red) and b* (positive value indicates yellow) values rise. The difference between two colors is a metric, ΔE in CIELab, that relates to the extent of the color change and can be calculated from the determined L*a*b* values with the following equation:

(1) $$\Delta E_{ab}^{*}=\sqrt{{\left(L_{2}^{*}-L_{1}^{*}\right)}^{2}+{\left(a_{2}^{*}-a_{1}^{*}\right)}^{2}+{\left(b_{2}^{*}-b_{1}^{*}\right)}^{2}}$$

The ΔE values (average of 10 pellets) were plotted over the API content (% *w*/*w*), and the scatter plot is shown on Figure 2B. Pellet size analysis was also carried out (Image Pro Plus; Media Cybernetics, Bethesda, MD, USA) and the mean Feret diameters are shown in the box plot of Figure 2C.

Another method to characterize the film coating thickness of pellets with image analysis was introduced by Andersson et al. The ethylcellulose-coated pellets were cut in the equator with a microtome blade and were photographed with a fluorescence microscope coupled with a digital camera. The manual analysis was based on the different fluorescence of the core and the coating [59]. Laksmana et al. used confocal laser scanning microscopy (CLSM) to visualize the coating layer in a non-invasive way. They added carmoisine as a fluorescent marker to the hydroxypropyl methylcellulose (HPMC) before coating and acquired images with an inverted immersion microscope to study the effect of process conditions (e.g., spraying rate, nozzle position) on coating thickness and its distribution. This method provides a highly automatable approach for the characterization, thus enabling the optimization of pellet coating parameters [60]. Coating also affects the pellet swelling properties. Kállai et al. placed polymethacrylate-copolymer-coated pellets with diverse cores into aqueous media of different osmolality. After shaking they used image analysis to study the shape parameters to gain information about the influence of coating permeability on swelling [61].

#### 2.1.5. Solid Microcapsules, Microspheres

Microcapsules are small (1–1000 μm) particles that consist of at least one core substance and a surrounding polymeric capsule wall. They are extensively used, for example, for taste masking, solubility enhancement, sustained or controlled delivery purposes, or even cell encapsulation [49]. Depending on their preparation, microcapsules may be solid in shape but may differ in wet state [62].

Confocal laser scanning microscopy is a versatile tool in the characterization of these microcapsules. The use of different suitable fluorescent labels for the oil and polymer phases enables the calculation of the volumes of the different phases after analyzing the respective confocal fluorescent pictures. As these pictures allow for the differentiation of the oil phase and the encapsulated air, the nondestructive determination of the oil encapsulation rate is also accomplishable with image analysis [63]. Similarly, the polymer ratios can be analyzed quantitatively as the CLSM grants insight below the surface, and the fluorescence signals correlate with the number of labeled substances in the microcapsule [64].

#### 2.1.6. Solid Self-Emulsifying Drug Delivery Systems (SEDDS)

Pelletization is not exclusively for solids, but applicable, for example, in the case of emulsion-based oral drug delivery systems like self-emulsifying drug delivery systems. They can spontaneously form micro- or nanoemulsions in the aqueous media of the gastrointestinal tract, and thus enhance the oral bioavailability of poorly water-soluble drugs [65,66]. To overcome the difficulties of the conventional emulsion dosage form, solid self-emulsifying formulations have also been developed [67,68]. The quality attributes of the thereof formulated pellets can be examined with both atomic force microscopy [67] and optical microscopy. Abdalla et al. analyzed a thousand pellets and determined that the aspect ratio of the self-emulsifying pellets was sufficient to ensure optimal pellet flow properties [68]. Solid SEDDS can also be formulated by suspending Aerosil 200 and spray drying the suspension. This method was employed by Balakrishnan et al., who used scanning electron microscopy and image analysis for the morphological characterization of the solid SEDDS particles [69].

#### 2.1.7. Nanofibers

Owing to their outstanding physicochemical characteristics (e.g., large surface area to volume ratio, stiffness and tensile strength), nanofibers are widely researched one-dimensional nanomaterials with a great potential for pharmaceutical and medical applications. Nanofiber fabrication can be accomplished by drawing, phase separation, template synthesis, etc., but electrospinning is probably the most generally adopted technology [70,71]. To analyze the morphology of the AC electrospun nanofibers from SEM images, coating is required to increase the sample conductivity. The application of gold coating is the norm, and the determination of fiber uniformity and individual fiber diameter data for statistical description and comparison is a standard method for morphological studies [72,73]. He et al. applied a gold–palladium alloy coating and determined not only the diameters of the 100 randomly chosen fibers but also their orientation by performing fast Fourier transform analyses [74].

Balogh et al. evaluated the distribution of the fiber diameters with a self-developed image analysis algorithm based on the SEM images. Their algorithm measured the diameters along randomly placed points on the SEM images so the arbitrary determination of the measurement location by the operator could be avoided. On each of the ten SEM images of the fibrous samples, ten diameters were measured (100 diameters per sample) [75].

#### 2.1.8. Films

To ease—mainly the pediatric and geriatric—drug administration, the number of attempts for the development of innovative drug delivery systems is endless. Orally disintegrating (or orodispersible) films are considered to be safe, effective, and modern solutions with great patient acceptance and are associated with numerous other advantages [76].

Redfearn et al. studied orodispersible films in an in vitro oral model where image analysis was used to determine the volume reduction of the film matrix over time, which well describes the disintegration behavior of the film [77].

#### 2.1.9. Tablets and Capsules

**Figure 3:**
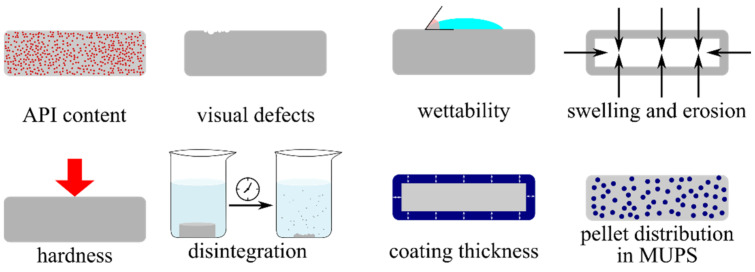
Examples for the use of image analysis in the characterization, evaluation, and quality control of tablets.

Oral administration is the most common drug delivery route where capsules and tablets are predominantly found. Due to numerous reasons, such as cost, ease of handling and packaging, or acceptance, tablets are preferred in general [49]. The pharmacopoeias describe several methods for the processing and quality control of tablets, but the extensively applicable image analysis methods are not yet regulated [1,2,3]. In this section a couple examples will be shown on the relevance of image analysis in tablet investigations (Figure 3).

A basic requirement for all dosage forms is the uniformity of contents of active ingredients that should be within a narrow range around the label claim [1]. Soponar et al. quantitatively evaluated the paracetamol and caffeine content of six different tablets with image analysis and reversed-phase high-performance thin-layer chromatography. After the component eluation, the chromatograms were visualized under UV light (λ = 254 nm), digitally recorded and analyzed. The authors claim their method was simple, rapid, precise, and accurate for the simultaneous quantitative evaluation of paracetamol and caffeine [78]. In the case of a colored substance, image analysis can be used for the prediction of its content. Mészáros et al. used meloxicam, a pastel yellow API and UV illumination to visualize the drug content. They compared the results of the used MV-based method to Raman chemical mapping and found it not only being suitable but also significantly quicker (ms vs. 18 h/tablet) [17].

**Figure 4:**
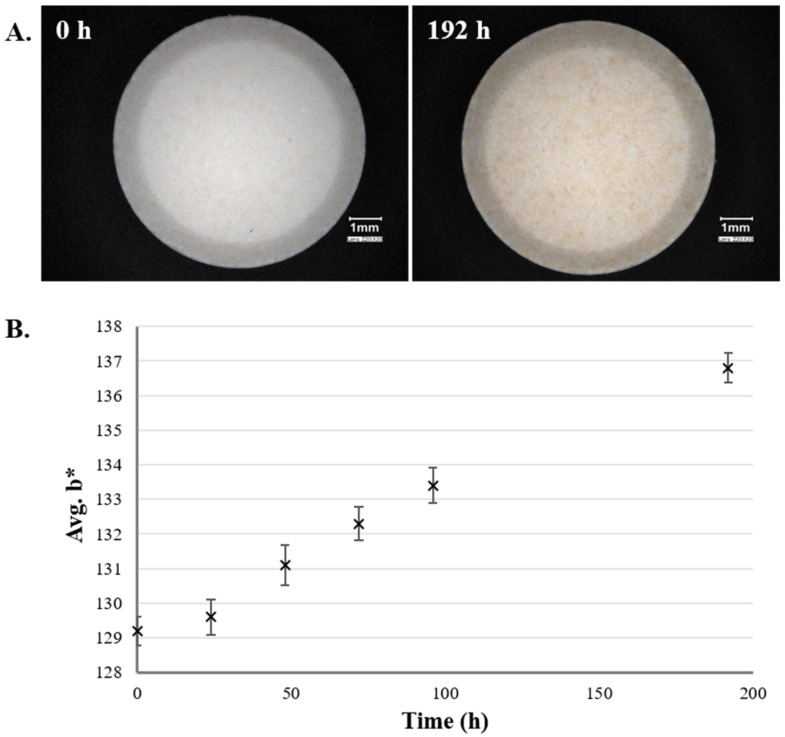
(**A**) Color change of tablets stored at 40 °C, 75% relative humidity and (**B**) the color analysis results (*n* = 10; Avg. ± SD evaluation of own experimental data).

The importance of appropriate storage conditions has long been known. Temperature, moisture, light, and ventilation are just some factors that need to be considered, as, among others, they highly influence the stability of tablets [79]. According to the European Pharmacopoeia, the specific storage conditions are determined by the competent authorities, the pharmacopoeial information and recommendations are mainly for the prevention of contamination and deterioration [1]. Image analysis can be applied as a brief evaluation technique for the color change of tablets stored under different conditions. Figure 4A shows tablets of the same batch stored for up to 192 h in 40 °C and 75% relative humidity. As in Section 2.1.4, the change in the color was analyzed from digital microscopic (Keyence VHX-970F; Keyence Corp., Japan) pictures (2048 × 1536 pixels; 10 photo/sample) and the L*a*b* color space was used for the numeric description of the yellowness. As expected, the rise in the value of b* (positive value indicates yellow) was parallel with the increasing storage time (Figure 4B).

During the tableting and packing procedures, problems can occur that result in tablets with defects—visual, or technological. Visual defects of tablets can be observed and quantified with the help of IA. Mollereau et al. carried out image texture analyses on different binary mixtures containing tablets and extracted numerically relevant data on the level of the defects [80].

Wettability is an essential property of tablets that influences dissolution and disintegration characteristics as these processes only start when the tablet comes into contact with the dissolution medium [81]. For characterization, direct or indirect contact angle measurements are commonly used. By the pharmacopeial definition, the contact angle is the angle that is naturally formed at the liquid–solid interface [1]. Wetting properties were studied by Csobán et al., who used image analysis to determine the contact angle via the dynamic sessile drop method. They used the information to evaluate the changes in the physical and physicochemical properties of tablets that were manufactured with different initial tensile strengths and thermally treated afterward to improve the mechanical properties of pellet-containing tablets [82].

Contact with water is the prerequisite of swelling. The swelling kinetics of diltiazem-containing cross-linked amylose tablets were studied by Moussa et al., who analyzed axial and radial swelling kinetics on transversal tablet sections after gravimetrically measuring the water uptake. They characterized the swelling as a percentage of the original size via IA and confirmed the higher the degree of cross-linking, the greater the increase is in the tablet diameter [83]. Swelling and erosion were investigated on cylindrical pure hydroxypropyl methylcellulose tablets by Chirico et al. They carried out long-lasting experiments where only radial water uptake was allowed and studied the progress for up to one week. An unexpected phenomenon was documented as a circular halo intermediate was observable between the erosion and the swelling radius [84].

The hardness of the tablets can affect many other important properties such as the disintegration time and/or the drug release profile. May et al. used terahertz pulsed imaging (TPI), a non-destructive technique usually used for quality control purposes, to determine the hardness of pharmaceutical solid dosage forms [85]. Mészáros et al. found that the surface of tablets made with low compression force was rougher and more porous while the surface of tablets made with the same composition but a high compression force was smoother. This difference was measurable via image analysis (machine vision) of the digital images taken from tablets, and the hardness became predictable [17].

Tablet disintegration can also be examined with image analysis, as shown by Berardi et al. They analyzed the behavior of single tablets in distilled water under controlled circumstances and took horizontal photos to explore the disintegration kinetics and mechanisms along with the effect of disintegrant concentration [18].

Modified release of tablets is often ensured by the application of coating. For the determination of coating thickness of tablets, image analysis is frequently applied. Šašić chiseled several tablets, imaged them, and used numerical pixel analysis to assess them statistically [86]. By contrast, Koller et al. choose a non-destructive method, optical coherence tomography, for the characterization of coating thickness throughout the spray coating process. This technique enables the study of the delicate coating layers at the beginning of the process [87].

Compressed multiunit pellet systems (MUPS) combine the advantages of tablets and multiparticulate dosage forms. They contain the active ingredient in several subunits that are combined into one system that contributes to excellent in vivo distribution, reduced local concentration, fewer side-effects, and consequently improved bioavailability. When it comes to compressed multiunit pellet systems, controlling the integrity of the subunits after the tableting process is essential. This can be accomplished with diversified methods. Mostly in vitro release studies are carried out or microscopic imaging techniques are used (alone or combined) with image analysis [14]. Wagner et al. used colored pellets and a repro camera for this purpose. Their method enabled the inspection only on the upper and lower surfaces of the tablet [88,89]. Multispectral UV imaging combined with image analysis has been reported as a fast and suitable method for pellet distribution analysis in MUPS tablets. The approach, which is applicable even in the case of coated tablets, was published by Novikova et al. [90]. In another study, they visualized the tablet–pellet interfaces to detect the coated pellets within the MUPS tablets with TPI [91]. Similarly, for the determination of the spatial distribution of potassium chloride pellets, Csobán et al. used another non-destructive method, microfocus X-ray (MFX) imaging. They supplemented the image results with conductivity measurements and found a linear relationship between the MFX-predicted and the actual KCl content. Furthermore, image analysis was also applied for compressed pellet shape characterization [20].

The analysis of the above-mentioned 3D image processing (micro-XRT) can also be used to examine capsules. Using a combination of quantitative image analysis and machine learning method, the authors identified and characterized the coated but damaged ibuprofen pellets within the capsule with very good accuracy using automatic detection [92].

There are numerous methods for altering the release profile of tablets to minimize side effects while enhancing the efficiency of the treatment and patient compliance. The osmotic pump-controlled release technique is a popular choice as it ensures prolonged effect and consistent blood concentration [93,94]. With altering the size of the drug release hole of osmotic tablets, the release kinetics can be significantly altered, thus the size must be controlled. For recognition and measurement purposes, machine vision can be applied, as shown by Zhang et al., who combined an edge detection algorithm with a Canny operator and used mathematical morphology with high precision [95].

Možina et al. developed a new method that enables the robust and real-time inspection of tablets in less than two ms. Their segmentation method tracks the object border and verifies the contour to segment the image of a single tablet and is easily applicable for various tablet designs. The approach is proposed to be independent of the tablet’s visual information, such as color, texture, prints, or engravings [96].

Even simple imaging equipment can be used to capture images of the right quality. Hirschberg and colleagues used a simple office scanner for tablet coating quality control. According to their results, using an appropriate algorithm, the tablets could be classified according to the perfection of the coating [97].

To address the increasing need for personalized drug administration, 3D printing is a potentially revolutionary method. This is supported by the fact, that the first 3D printed tablet, Spritam^®^ is already on the market [98]. Water-based 3D inkjet printing is an additive manufacturing method where the importance of the generated droplet size is of great importance. It affects the volume and consequently the dose of API as well [99].

**Table 2:** Image analysis for the characterization and evaluation of solid dosage forms.

Dosage Form	Aim of Measurement	Image Acquisition Method	Software	Reference(s)
Powder (Micronized Bulk)	Particle Size	Optical Microscopy, SEM	Olympus Cue-2 Morphometry Software	[31]
Powder (Non-Spherical Particles)	Morphology	Optical Microscopy	N/A	[32]
Powder	Morphology, Morphometry	Digital Camera, Optical Microscopy	N/A	[33]
Powder	Homogeneity	Digital Camera	ImageJ; N/A	[34,35]
Powder for Inhalation (DPI)	Aerodynamic Particle Size	Digital Camera	Matlab	[41,42]
Granulates	Morphology, Morphometry	Qicpic Dynamic IA System	Qicpic Dynamic IA System	[45]
Wet and Dry Dispersion	Size Distribution, Particle Brittleness, Tendency to Agglomerate	Optical Microscopy	Imagepro	[100]
Pellet	Form Factors	Optical Microscopy; Digital Camera	Pcimage VGA 24, Imagepro Plus, Sigmascan Pro Image Analysis; Seescan Sonata, ImageJ	[14,20,29,82]
Pellet	Size, Shape	Stereomicroscope	Quantimet 500	[101]
Pellet	Particle Size Distribution	Retsch Camsizer Particle Size Analyzer	Retsch Camsizer Particle Size Analyzer	[50]
Pellet	Form Factors	Optical Microscopy	PC Image, Sigmascan Pro Image Analysis	[52]
Pellet	Coating Thickness	N/A	Morphologi™ G2 Image Analysis System	[55]
Pellet	At-Line Monitoring of Coating	Digital Camera	Matlab, Image Processing Toolbox	[56]
Pellet	In-Line Monitoring of Coating	Digital Camera	N/A	[57]
Pellet	Coating Thickness	CLSM	ImageJ	[58]
Pellet	Coating Thickness	Digital Camera	Adobe Photoshop, Image Pro Plus	[21]
Pellet	Coating Thickness	Fluorescent Microscopy; CLSM + Optical Microscopy	Matlab Image Processing Toolbox	[59,60]
Pellet	Coating Uniformity	Office Scanner	In-House Program	[102]
Pellet	Porosity, Pore Size Distribution	CLSM + Optical Microscopy	Matlab Image Processing Toolbox	[60]
Pellet (Coated)	Swelling	Optical Microscopy	Image Pro Plus	[61]
Microparticles	Encapsulation Rate, Polymer Ratio in the Wall	Confocal Laser Scanning Microscopy	Scion Image	[63,64]
Self-Emulsifying Pellet	Pellet Shape (Feret Diameter)	Optical Microscopy	Image C	[68]
Solid Self-Emulsifying Particles	Particle Size	Scanning Electron Microscopy	Imageinside	[69]
Nanofibers	Fiber Diameter	Scanning Electron Microscopy	ImageJ, N/A	[72,73,74,75]
Films	Volume Reduction of the Film Matrix Over Time	Video Camera	Matlab	[77]
Tablet	API Content	Digital Camera	Sorbfil TLC	[78]
Tablet	(Colored) API Content	Digital Camera	Machine Vision (Matlab)	[17]
Tablet	Defects	Digital Camera	Visilog 6.8	[80]
Tablet	Wetting Properties	Digital Camera	ImageJ	[82]
Tablet	Swelling Kinetics	Video Camera	Ultimage™/X	[83]
Tablet	Swelling and Erosion (Water Mass Fraction)	Video Camera, Digital Camera	Mathcad^©^	[84]
Tablet	Swelling	USB-Microscope	ImageJ	[103]
Tablet	Hardness	TPI	N/A	[85]
Tablet	Hardness	Digital Camera	Machine Vision (Matlab)	[17]
Tablet	Disintegration	Digital Camera	ImageJ	[18]
Tablet	Coating Thickness	Near-Infrared Mapping Microscope	Matlab	[86]
Tablet	Coating Thickness	Optical Coherence Tomography	N/A	[87]
MUPS Tablet	Pellet Distribution at the Surfaces	Reprocamera	Image Pals 2 GO	[88,89]
MUPS Tablet	Tablet–Pellet Interface	UV Imaging	Matlab, PLS_Toolbox, Image Processing Toolbox	[90]
MUPS Tablet	Pellet Distribution	TPI, Multispectral UV Imaging; Microfocus X-ray Imaging	Mathlab; N/A	[20,91]
Capsule	Broken Coated Pellets	Micro-XRT	Matlab	[92]
Tablet	Visual Tablet Inspection	N/A	Borland C++ Builder	[96]
Tablet	Coating Quality	Office Scanner	Artificial Intelligence	[97]
3D-Printed Tablet	Droplet Size	Drop Watcher of Fujifilm Dimatix Materials Printer DMP-2850 Series	ImageJ	[99]
3D Printlets	Difference Characterization	Raspberry Pi Camera	Custom-Developed Software	[104]
Biopolymer Coating	Wettability, Contact Angle	Digital Camera	SCA20	[105]

### 2.2. Semisolid Formulations

#### 2.2.1. Ointments and Creams

Creams and ointments are semisolid formulations. Both the single-phase ointments and the multiphase (consisting of an aqueous phase and a lipophilic phase) creams are for various administration routes. They are not only used for cutaneous purposes but also for ocular, intrauterine, vaginal, and rectal administration [1].

Dong et al. studied cyclosporine-containing ophthalmic ointments and used image analysis for the examination of quality attributes. They worked with a polarized light microscope and ImageJ software, and by comparing them to a blank sample they ascertained the dissolved state of the active ingredient [106].

The influence of processing conditions on the characteristics of the preparations is a well-known fact. In the case of an oil-in-water (O/W)-type emulsion cream, the technology affects the dispersion level of the internal phase, the rheological properties, and also the drug availability among other factors. Realdon et al. used optical microscopy linked to computerized image analysis for the exploration of the differences in the level of dispersion of the inner oily phase. They studied over 500 droplets per batch and determined the frequency of size classifications [107,108]. Sutton et al. worked with a polarized light microscope to investigate the effect of pH on the physical stability of the liquid crystal-stabilized O/W-type emulsion cream. They determined the droplet size distribution of the inner phase and found that both the pH and the degree of emulsifying way neutralization are influential parameters [109].

#### 2.2.2. Suppositories

Although better and more robust methods are available, the naked eye is still often used for visual inspection. Saleem et al. evaluated tramadol-hydrochloride-containing rectal suppositories visually for surface evenness after having them longitudinally cut [110]. Patil et al. loaded flutamide-bearing mucoadhesive microparticles into poloxamer suppositories and used image analysis for the evaluation of the microparticle sizes [111].

**Table 3:** Image analysis for the characterization and evaluation of semisolid dosage forms.

Dosage Form	Aim of Measurement	Image Acquisition Method	Software	Reference(s)
(Ophthalmic) Ointment	API Particles in the Ointment	Polarized Light Microscopy	ImageJ	[106]
Cream (O/W)	Droplet Size of the Dispersed Phase	Optical Microscopy	Casti Studio Imaging	[107,108]
Cream (O/W)	Droplet Size of the Dispersed Phase	Polarized Light Microscopy	Malvern Morphologi G3	[109]
Rectal Suppository	Surface Evenness	Visual Inspection	-	[110]
Microparticle-Loaded Suppository	Microparticle Size	Digital Microscope	Motic Images 2000	[111]

### 2.3. Liquid Formulations

#### 2.3.1. Solutions (Eye Drop)

Surface tension (ST) has a larger influence on the pharmaceutical manufacturing process than one might think. Further to the typical examples of emulsions and suspensions, it plays an important role in the case of eye drops or even solid dosage forms [112]. Grgurević et al. highlighted the importance of the ST of eye drops and proposed a method based thereon for the evaluation of compatibility of the tear fluid and the eye drops to avoid eye-related discomfort [113]. The pendant drop method [114] can be easily combined with video imaging and image analysis systems for surface tension determination [115].

A novel use of image analysis was published by Berasarte et al., whose method enables the simultaneous determination of the pH and phosphates in eye drops. Their method is based on the automatic quantification of color change. In the case of phosphates, a blue molybdenum complex is formed in a two-step reaction, depicted in Equations (2) and (3), and as known from the Lambert–Beer law, the intensity of color (absorbance) is proportional to the analyte concentration.

(2) $$PO_{4}^{3-}+12MoO_{4}^{2-}+27H^{+}\to H_{3}PO_{4}{\left(MoO_{3}\right)}_{12}+12H_{2}O$$

(3) $$H_{3}PO_{4}{\left(Mo^{VI}O_{3}\right)}_{12}+reductant\to {\left[H_{4}PMo_{8}^{VI}Mo_{4}^{V}O_{40}\right]}^{3-}$$

For the determination of the pH, bromothymol blue indicator was chosen. The results were compared to reference methods, and as the differences were non-significant the method is claimed to be suitable along with being simple, quick, sensitive, and economical [116,117].

#### 2.3.2. Emulsions

Emulsions are dispersed systems containing at least two immiscible liquid phases where the inner phase is dispersed in the outer phase in the form of droplets [49].

To overcome the limitations of manual quality assessment of emulsions, machine learning is a novel method for pharmaceutical industries. In situ, automated droplet detection and size measurements in emulsion systems have already been used to understand and control the emulsification process [118,119,120]. Unnikrishnan et al. implemented the technology to the pharmaceutical manufacturing of an emulsion cream. They followed the process with an optical microscope and used an automated image processing macro to gain information on droplet characteristics. Comparing the results to manual analysis they found the automated method not only significantly faster but also more accurate and repeatable [121].

Bright-field optical microscopy can be applied to analyze the droplet size distribution of emulsions. Reufer et al. found that with the increasing viscosity of the xanthan gum-bearing outer phase, the dispersed almond oil droplets became more uniform. They also noted that due to the limitations of optical microscopy, the detection of submicron size droplets is not accomplishable [122].

Although the pharmaceutical use of particle-stabilized emulsions is yet uncommon, they could be used as controlled-release drug delivery systems [123]. Prestidge et al. coated emulsion droplets with modified silica nanoparticles and with the help of image analysis they studied the coalescence kinetics. They also adsorbed different nanoparticles and investigated their influence on droplet stability. Even though laser diffraction is a wholly common method studying emulsion droplet size, unlike image analysis it cannot give information about the flocculation or droplet coalescence [124,125].

#### 2.3.3. Suspensions

Suspensions are dispersed systems where the internal, solid phase is uniformly dispersed in the liquid medium. The amount and particle size of the suspended ingredient are of great importance as they influence not only the drug solubility, the product appearance and the re-suspendability, but also the in vivo absorption, and the overall stability [49].

Ciprofloxacin-containing mucoadhesive suspensions were examined by Sahoo et al., who used scanning electron microscopy to analyze the particle surface and shape. To avoid incorrect edge detection of the particles, they analyzed manually and used software to gain particle size distribution and aspect ratio data. They correlated the results with the mechanical properties and concluded that the presence of carbopol contributes to the uniform dispersion of particles and the increased stability [126].

Kollidon^®^ SR is usually used for pH-independent sustained-release matrix tablets, but Arias et al. formulated a colloidal suspension as an alternative drug delivery system. Image analysis served as a supplementary tool for the goniometer to measure contact angles to evaluate the wettability of the solid [127].

#### 2.3.4. Aerosols

The importance of the aerodynamic assessment of fine particles is supported by the fact, that the pharmacopoeias describe distinct methods for their determination based on the type of the inhalers employed [1,2,3]. The size of aerosol particles can be determined via image analysis. Hallworth et al. used Quantimet^®^ 720, an image-analyzing computer from the latter part of the 1970s. With the machine’s help, they compared the particle size of suspension-type steroid-containing metered aerosols and examined the effect of firing position on particle distribution [128].

#### 2.3.5. Foams

Based on the conventional classification, foams are colloid systems that consist of a discontinuous gas phase that is dispersed in the continuous liquid or solid phase in the form of bubbles [129].

The use of medicated foams is not yet as common as other conventional dosage forms, so image analysis of pharmaceutical foams has not yet been extensively investigated. More articles can be found on the topic in other disciplines. Kennedy et al. applied IA to quantify the bubble coarsening dynamics in firefighting foams [130], but it is also widely used in food engineering. The microstructure of food is essential as it influences the density, texture, digestibility, and appearance as well as contributes to the flavor intensity and mouthfeel. Foams and aerated foods can be consumed as parts of beverages, baked goods, and cereal-based products as well as dairy or confectionery products [131,132,133]. Foam formation and stability were investigated on protein solutions by Guillerme et al., who used the correlation between electric resistance and liquid quantity along with video image analysis for the inspection of foam evolution by determining the level of foam in the device and the quantity of liquid at the bottom [134]. Furthermore, Krüss developed a dynamic foam analyzer for the scientific analysis of foams. The device enables the study of the foam structure (foam height, density, bubble size distribution, and its change over time) and the liquid content (foam drainage, stability, and determination of half-life) [135].

In our previous works, we analyzed images of foams to gain information on microscopic properties (shape, size, and size distribution of bubbles) that influence the macroscopic attributes of foams [136,137]. Furthermore, we used image analysis to characterize foam collapse, an essential parameter describing foam stability. By studying the formation kinetics of a single bubble from a dilute solution, we deuced therefrom to foam formation kinetics and macroscopic attributes [137].

**Figure 5:**
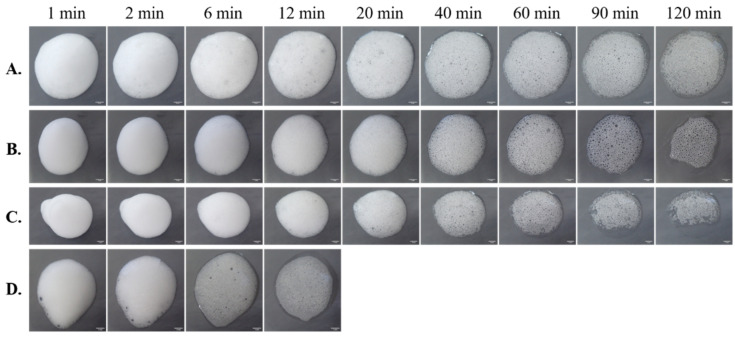
Comparison of foam spread and decay over time in cases of compositions with different surface-active agents ((**A**) sodium lauryl sulfate, (**B**) Tween 80, (**C**) Tween 20, and (**D**) Labrasol^®^; own experimental data).

A major advantage of the application of foams is easy spreadability, even on large hirsute areas, without greasy residue. Foams can be categorized into three classes based on their breakability, the breakdown or collapse after actuation: breakable, quick breaking, and stable [138]. The tendency of foams to collapse after actuation is well demonstrated in Figure 5, where the foam collapse and decay processes of four different foam compositions (sodium lauryl sulfate (SLS), Tween 80 (T80), Tween 20 (T20), and Labrasol^®^ (L)) were followed using a digital camera (Olympus Stylus TG-4 digital camera; Olympus Corp., Shinjuku, Tokyo, Japan).

**Figure 6:**
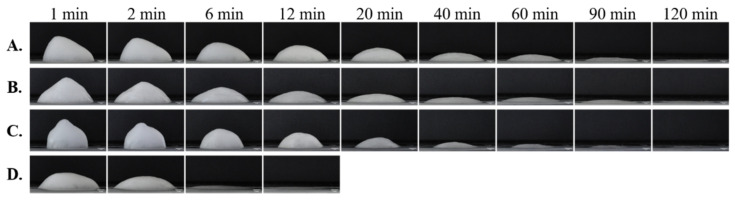
Evaluation of the foam collapse process in cases of compositions with different surface-active agents ((**A**) sodium lauryl sulfate, (**B**) Tween 80, (**C**) Tween 20, and (**D**) Labrasol^®^; own experimental data).

The foam decay progress can be studied also from horizontally taken pictures (Nikon D90 with Nikon AFS-50 mm f/1.8 G objective, Nikon Corp., Japan; Figure 6).

**Figure 7:**
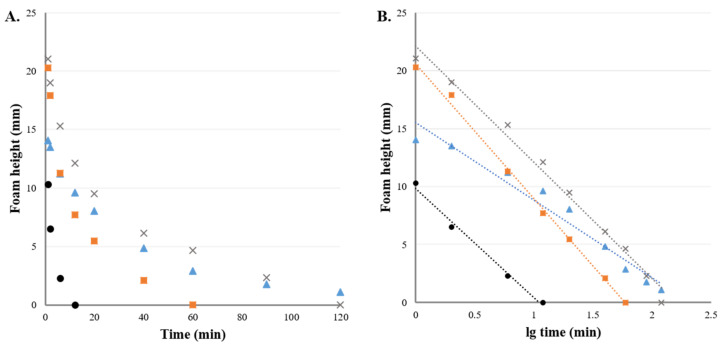
Analysis of foam heights (**A**) and collapse kinetics (**B**) of compositions with different surface-active agents (cross–sodium lauryl sulfate, triangle–Tween 80, square–Tween 20, circle–Labrasol^®^) with fitted trendlines (dotted lines) (evaluation of own experimental data).

By analyzing the distance between the foam base and the highest point, the height change can be quantified. If the foam height is plotted over time, foam collapse is easily comparable (Figure 7A). In addition, if time is logarithmized, the kinetics can be studied (Figure 7B). The equations of the fitted trendlines for the different surface-active agents, sodium lauryl sulfate (Equation (4); R = 0.9946), Tween 80 (Equation (5); R = 0.9811), Tween 20 (Equation (6); R = 0.9986), and Labrasol^®^ (Equation (7); R = 0.9950), are shown below.

(4) $$y_{SLS}=-10.062x+22.164$$

(5) $$y_{T80}=-6.6987x+15.529$$

(6) $$y_{T20}=-11.649x+19.935$$

(7) $$y_{L}=-9.4239x+9.8506$$

The data suggest that the foam height at any time (*F_t_*) can be calculated if the initial height (*F_0_*) and the speed constant (*k*) for the given composition is known by the following correlation equation:

(8) $$F_{t}=F_{0}\times e^{-k\times t}$$

**Table 4:** Image analysis for the characterization and evaluation of liquid dosage forms.

Dosage Form	Aim of Measurement	Image Acquisition Method	Software	Refs.
Solution (Eye Drop)	Phosphates, pH	Digital Camera	Matlab	[116]
Emulsion Cream	Droplet Characterization (Size, Shape)	Optical Microscopy	Fiji Based Machine Learning	[121]
Emulsion (O/W)	Droplet Size Distribution	Bright Field Microscopy	ImageJ	[122]
Emulsion	Droplet Coalescence Kinetics, Influence of Adsorbed Particles	Optical Microscopy	Optimas Image Analysis System	[124,125]
Suspension	Wettability (Contact Angle)	Digital Camera	N/A	[127]
Aerosol, Suspension	Particle Size	Quantimet^®^ 720	Matlab	[128]
Foam	Foam Decay, Bubble Size, Shape	Digital Camera	ADVANCE	[135]
Foam	Microscopic Properties, Foam Collapse	Digital Camera	ImageJ	[137]

## 3. Conclusions

Nowadays, the number of possibilities to digitally image and analyze dosage forms using various imaging procedures is endless. This summary illustrates that, regardless of the state of the drug product (liquid, semisolid or solid), the use of image analysis is manifold—ranging from the simplest methods (such as evaluation with the naked eye) to the more complex, MV-based techniques. The importance of these analysis tools lies in the applicability, not only during the development procedure as an in-process control tool, but also for process analysis (PAT).

Shape and size, along with its distribution, are the most studied parameters as they highly influence solubility, dissolution from the dosage form, and bioavailability. Furthermore, the modern image analysis software allows for the quick measurement of angles and thicknesses. Information can be gained about the physicochemical properties of the dosage forms, like wetting of a tablet or suspension, which ultimately affects the release of the API. Moreover, with the properly chosen method, the interior of the dosage forms can be mapped nondestructively in three dimensions, which allows for quantitative examinations. This is of extreme importance, e.g., in cases of multiparticulate dosage forms, as the constituting units can be damaged during tableting or capsule filling, and due to these structural defects, decrease in the therapeutic effect or even unwanted side effects may occur.

Further to the advantages of IA, in cases of dosage forms that are not yet highly regulated by the pharmacopoeias, like foams, its application remarkably broadens the yet limited toolkit for characterization and evaluation.

All of this highlights the importance of choosing the right image acquisition method and analyzing digital images of the right quality while the process can be simplified and accelerated when used in combination with machine learning.

## Figures and Tables

**Table 1:** A brief overview of the advantages of image analysis over a few conventional techniques.

Parameters	Conventionally Used Techniques	Advantages of the Image-Based Techniques over the Commonly Used Ones
Particle Size Distribution (PSD)	Sieve Analysis	Simultaneous Investigation of Particle Shape [16] Real-Time Results Rapidly
API Content	HPLC Mass Spectrometry UV-VIS Spectrometry	Nondestructive Ecofriendly (No Chemicals) Quicker and Easier Sample Preparation (if Needed)
Tablet Hardness	Tablet Hardness Tester	Nondestructive [17]
Disintegration of Tablets	Disintegration Apparat A (Ph.Eur.)	Kinetics and Mechanism can be Studied [18]
Pellet Uniformity in MUPS	Manual	Internal Defects can also be Detected [19] Nondestructive [20] Quicker
Coating Level of Dosage Forms	In Vitro Dissolution Testing	Not Necessarily Destructive Quicker Cost-Effective [21]
